# Sequential sampling model for multiattribute choice alternatives with random attention time and processing order

**DOI:** 10.3389/fnhum.2014.00697

**Published:** 2014-09-09

**Authors:** Adele Diederich, Peter Oswald

**Affiliations:** ^1^Cognitive Psychology, School of Humanities and Social Sciences, Jacobs UniversityBremen, Germany; ^2^Mathematics, Modeling, and Computing Center, School of Engineering and Science, Jacobs UniversityBremen, Germany

**Keywords:** sequential sampling, multiattribute, attention time, time schedule, order schedule, finite time horizon, Ornstein-Uhlenbeck, Wiener

## Abstract

A sequential sampling model for multiattribute binary choice options, called *multiattribute attention switching* (MAAS) model, assumes a separate sampling process for each attribute. During the deliberation process attention switches from one attribute consideration to the next. The order in which attributes are considered as well for how long each attribute is considered—the attention time—influences the predicted choice probabilities and choice response times. Several probability distributions for the attention time with different variances are investigated. Depending on the time and order schedule the model predicts a rich choice probability/choice response time pattern including preference reversals and fast errors. Furthermore, the difference between finite and infinite decision horizons for the attribute considered last is investigated. For the former case the model predicts a probability *p*_0_ > 0 of not deciding within the available time. The underlying stochastic process for each attribute is an Ornstein-Uhlenbeck process approximated by a discrete birth-death process. All predictions are also true for the widely applied Wiener process.

## 1. Introduction

Sequential sampling models are powerful models to account simultaneously for choice probabilities and choice response times. They have become the dominant approach to modeling decision processes in cognitive science. Their application includes a variety of psychological tasks from basic perceptual decision to complex preferential choice tasks. Early on they have been applied to identification and discrimination tasks (e.g., Edwards, 1965; Laming, 1968; Pike, 1973; Link and Heath, 1975; Heath, 1981; Ashby, 1983); memory retrieval (e.g., Stone, 1960; Ratcliff, 1978; Van Zandt et al., 2000); and classification (e.g., general recognition theory, Ashby, 2000; exemplar–based random walk models of classification, Nosofsky and Palmeri, 1997) to account for speed-accuracy data. They have also been used for preferential decision tasks (e.g., decision field theory (DFT), Busemeyer and Townsend, 1993; multiattribute dynamic decision model, Diederich, 1997; Diederich and Busemeyer, 1999) to account for choice response times and choice probabilities interpreted as preference strength; judgment and confidence ratings (Pleskac and Busemeyer, 2010); to account for selling prices, certainty equivalents, and preference reversal phenomena (Busemeyer and Goldstein, 1992; Johnson and Busemeyer, 2005). More recently, they have been applied to combining perceptional decision making and payoffs (Diederich and Busemeyer, 2006; Diederich, 2008; Rorie et al., 2010; Gao et al., 2011). Furthermore, these models have been closely linked to measures from neuroscience like multi-cell electrode recordings (e.g., Ditterich, 2006; Gold and Shadlen, 2007; Churchland et al., 2008).

Sequential sampling models assume that (1) stimulus and choice alternative characteristics can be mapped onto a hypothetical numerical value representing the instantaneous level of evidence (activation, information, or preference—the wording often depends on the context), (2) some random fluctuation of this value over time occurs, (3) this evidence is accumulated over time, and (4) a final choice is made as soon as the evidence reaches a threshold. Therefore, sequential sampling can be described as a stochastic process. Two quantities are of foremost interest: (1) the probability that the process eventually reaches one of the thresholds or boundaries for the first time (the criterion to initiate a response), i.e., *first passage probability*; (2) the time it takes for the process to reach one of the boundaries for the first time, i.e., *first passage time*. The former quantity is related to the observed relative frequencies, the latter usually to the observed mean choice response times or the observed choice response time distribution.

Two classes of sequential sampling models have been predominantly used in psychology: Random walk/diffusion models and accumulator/counter models. The former are typically applied to a binary choice task, so that evidence for one choice alternative is at the same time evidence against the other. A decision is made as soon as the process reaches one of two preset criteria. In the latter, an accumulator/counter is established for each choice alternative separately, and evidence is accumulated in parallel. A decision is made as soon as one counter wins the race to reach one preset criterion. The accumulators/counters may or may not be independent. In the following we focus on random walk/diffusion models. For a review of both diffusion models and counter models see (Ratcliff and Smith, 2004).

**Figure 1:**
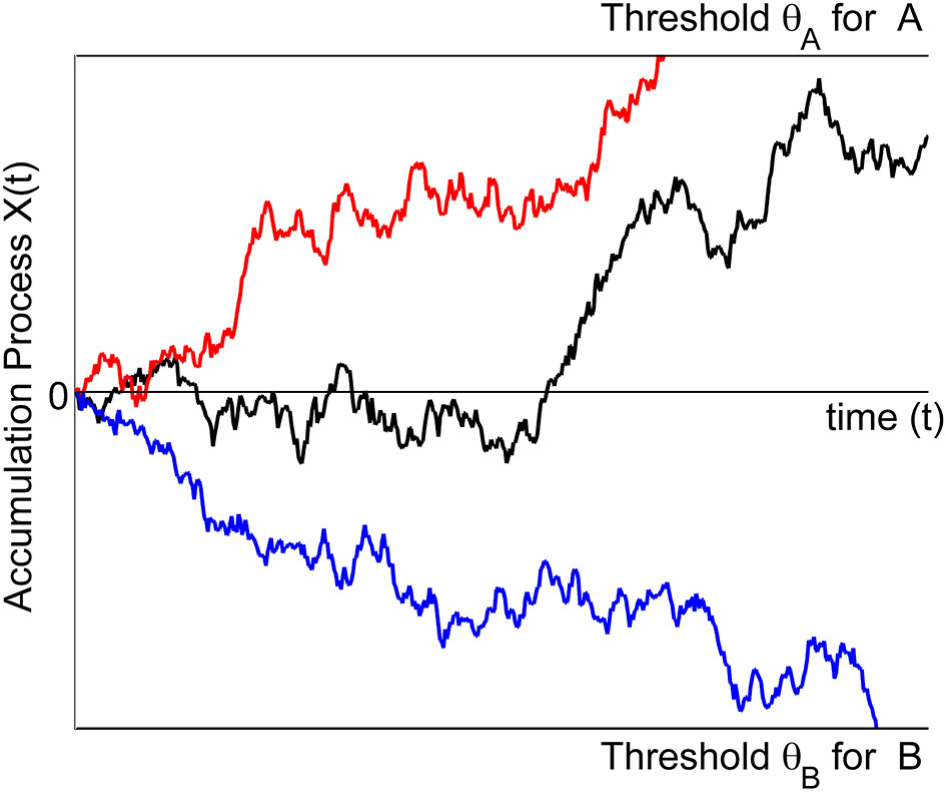
**The trajectories symbolize the accumulation process for three different trials.** In one trial (red) the process is absorbed at the boundary for making an A response. In another trial (blue) the process is absorbed at the boundary for making a B response. For the third trial (black) the accumulation process still evolves and no response is yet initiated.

To be more precise and to introduce notation, let *X*(*t*) denote the accumulation process. For a binary choice, say between choice options A and B (Figure 1), the models assume that the decision process begins with an initial state of evidence *X*(0). This initial state may either favor option A (*X*(0) > 0) or option B (*X*(0) < 0) or may be neutral with respect to A or B (*X*(0) = 0). Upon presentation of the choice options, the decision maker sequentially samples information from the stimulus display over time, retrieves information from memory, or forms preferences, depending on the context. The small increments of evidence sampled at any moment in time are such that they either favor option A (*dX*(*t*) > 0) or option B (*dX*(*t*) < 0). The evidence is accumulated from one moment in time to the next by summing the current state with the new increment: *X*(*t* + *h*) ≈ *X*(*t*) + μ(*X*(*t*), *t*) *h* + σ (*X*(*t*), *t*) (*W*(*t* + *h*) − *W*(*t*)). Here, μ(*x*, *t*) is called the *drift rate* and describes the expected value of increments per unit time. The factor σ(*x*, *t*) in front of the increments *W*(*t* + *h*) − *W*(*t*) of a standard Wiener process *W*(*t*) is called the *diffusion rate*, and relates to the variance of the increments. This process continues until the magnitude of the cumulative evidence exceeds a threshold criterion, θ. The process stops and option A is chosen as soon as the accumulated evidence reaches a criterion value for choosing A (here, *X*(*t*) = θ*_A_* > 0) or it stops and chooses option B as soon as the accumulated evidence reaches a criterion value for choosing B (here *X*(*t*) = θ*_B_* < 0). The probability of choosing A over B is determined by the accumulation process reaching the threshold for A before reaching the threshold for B. The criterion is assumed to be set by the decision maker prior to the decision task.

The Wiener process with drift, lately called *drift-diffusion model* in the psychological literature (Bogacz et al., 2006), is the most widely applied model. Different versions reflect additional assumptions for specific psychological domains. Ratcliff (1978) proposed a diffusion model for memory retrieval that is used for various psychological decision tasks. It is based on the work by Laming (1968) and Link and Heath (1975) and assumes variability in the starting point (i.e., *X*(0) follows a uniform distribution), and the drift rate μ = μ(*t*) of the Wiener process is normally distributed (cf. Laming). The residual time, i.e., the time other than the decision time, such as stimulus encoding and motor response, is assumed to be uniformly distributed and added to the decision time, i.e., response time equals the decision time plus a residual (non-decision) time. For a recent overview with applications see Voss et al. (2013). Other approaches include the Ornstein-Uhlenbeck model that linearly accumulates evidence with decay (Busemeyer and Townsend, 1993; Diederich, 1997), and the leaky competing accumulator model (Usher and McClelland, 2001) that non-linearly accumulates evidence with decay.

Common to almost all of these approaches is the assumption that a single integrated source of evidence generates the evidence during the deliberation process leading to a decision. In particular, the integrated source may be based on multiple features or attributes, but all of these features or attributes are assumed to be combined and integrated into a single source of evidence, and this single source is used throughout the decision process until a final decision is reached. Diederich (e.g., Diederich, 1995, 1997, 2003, 2008), however, assumed a separate process for each attribute^1^. The decision maker switches attention from one attribute to the next during the time course of one trial. For instance, in a crossmodal task (visual, auditory, tactile), Diederich (1995) assumed a serial processing controlled by stimulus input at given stimulus onset asynchronies (SOA). That is, the order of attributes, here a light, followed by a tone, followed by a tactile vibration, as well as the point in time when a new attribute was added, here the tone presented at *t*_1_ (*t*_1_ ms after the light onset) and the tactile vibration at *t*_2_ (*t*_2_ ms after the light onset) was determined externally by the experimental setup. In the following we will call an attention switch at predetermined, fixed times, and predefined order attributes, a *deterministic time and order schedule*. Often, however, neither the processing order of attributes nor the point in time when the decision maker switches attention from one attribute to the next are known or can be inferred from the experimental setup. For those cases, Diederich (1997) proposed a specific model in which attention switches from one attribute to the next with some probability. This is an instance of a *random time and order schedule* which will be investigated more systematically in the present study.

The purpose of this paper is to present a unified treatment of sequential sampling models for both deterministic and random time and order schedules. To do so we start with deriving expressions for mean choice response times and choice probabilities for a deterministic time and order schedule before we show how they extend to random time and order schedules, including Poisson, binomial, geometric, and uniform distributions for the attention time devoted to each attribute in the sequence before attention switches to the next randomly or deterministically chosen attribute. We will provide first numerical evidence on the influence of various properties of a schedule on the predictions for mean choice response times and choice probabilities.

## 2. Preliminaries

The model applies to any finite number of attributes that the decision maker may consider, i.e., *k* = 1, …, *K*. For convenience we first describe the process for one attribute. As underlying information process for each attribute we assume an Ornstein-Uhlenbeck process *X*(*t*) defined by

(1) $$dX(t)=\left(\delta_{k}-\gamma_{k}X(t)\right)dt+\sigma_{k}dW(t),$$

where *W*(*t*) is a standard Wiener process. The parameters δ*_k_*, γ*_k_*, and σ*_k_* are characteristics of the *k*-th attribute. The attribute characteristics may affect the quality of the extracted evidence for choosing *A* over *B* and this quality of evidence determines the drift rate δ*_k_*. That is, the better an attribute discriminates between *A* and *B*, the larger is δ*_k_*. The parameter γ*_k_* which induces a change of the drift rate depending on the current state in the state space is often connected to memory processes (e.g., primacy and recency effects), conflict situations (e.g., approach-avoidance), or similarities between choice alternatives. Thus, together the effective drift δ_*k*_ − γ_*k*_*X*(*t*) determines the direction and the velocity of the process when considering the *k*-th attribute at time *t*. Note that by setting γ*_k_* to 0 results in a Wiener process with drift. That is, all the analysis we perform in the following is also valid for the Wiener process with drift. The diffusion coefficient σ_*k*_ indicates the variance of the increments of the process, for simplicity, we will set σ*_k_* = σ for all *k*.

### 2.1. Matrix approach

Stochastic processes such as the above *X*(*t*) can be approximated by a discrete time, finite state space Markov chain. We use the matrix approach since it is simple to implement, sufficient in determining the entities of interest, i.e., choice probabilities and choice response times, and flexible to account for non-stationary and non-linear properties one wishes to include for the decision making process in the future. The continuous state space [θ*_B_*, θ*_A_*] of the piecewise Ornstein-Uhlenbeck process *X*(*t*) is replaced by a finite state space *S* = {−*m_B_*, …, *m_A_*} with *m* = *m_A_* + *m_B_* + 1 states. The diffusion process {*X*(*t*), *t* ≥ 0} is approximated by a discrete random walk {$\tilde{X}$(*n*), *n* ≥ 0} with values in *S* such that *X*(*n*τ) ≈ Δ · $\tilde{X}$(*n*) and θ*_A_* ≈ *m_A_*Δ and θ*_B_* ≈ −*m_B_*Δ, where Δ is the step size of change in evidence. To achieve convergence in the limit, the discretization parameters (Δ for state space, and τ for time) are tied to each other by the relation Δ = σ $\sqrt{\tau }$.

The attribute-related matrices *P_k_*, *k* = 1, …, *K*, are given in their canonical form by

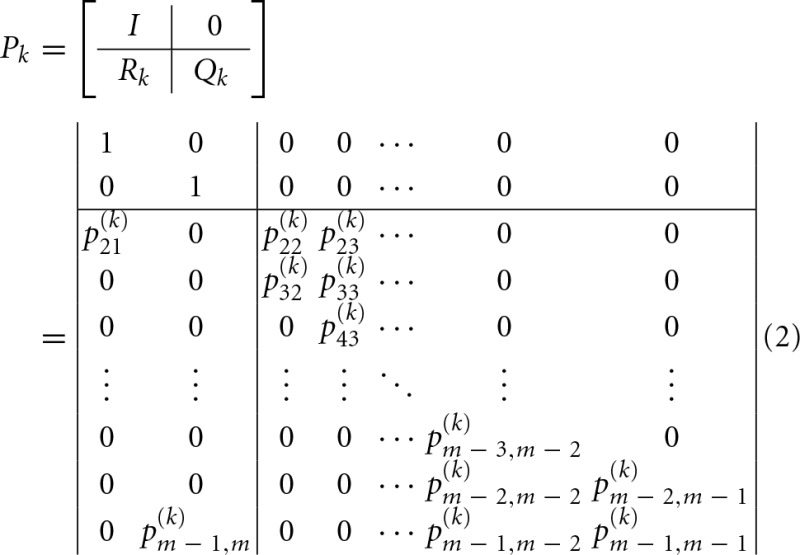



where

$$\begin{array}{l} p_{i,\;i-1}^{\left(k\right)}=\frac{1}{2}\left(1-(\delta_{k}-\gamma_{k}i\Delta )\frac{\sqrt{\tau }}{\sigma }\right),\\ p_{i,\;i+1}^{\left(k\right)}=\frac{1}{2}\left(1+(\delta_{k}-\gamma_{k}i\Delta )\frac{\sqrt{\tau }}{\sigma }\right), \end{array}$$

for *i* = 2, …, *m* − 1 (here, the index *i* corresponds to the state *i* − 1 − *m_B_*). As Δ → 0 (or, equivalently, τ → 0), the decision probabilities and mean choice response times obtained from the Markov chain model converge to the values obtained from the underlying continuous process *X*(*t*). The identity matrix *I* corresponds to the two absorbing states (−*m_B_* and *m_A_*) associated with the two decision thresholds, one for each choice alternative; the matrix *Q_k_* contains the transient probabilities, corresponding to the updating evidence process, and the matrix *R_k_* contains the one-step transition probabilities from the transient to the absorbing states. In particular, the first column vector of the matrix *R_k_* (denoted by *R*_*B*,*k*_) contains the transient probabilities for reaching alternative *B*, while the second *R*_*A*,*k*_ contains the ones for alternative *A*. For details and derivations see Diederich (1997) and Diederich and Busemeyer (2003).

### 2.2. Time and order schedule

**Figure 2:**
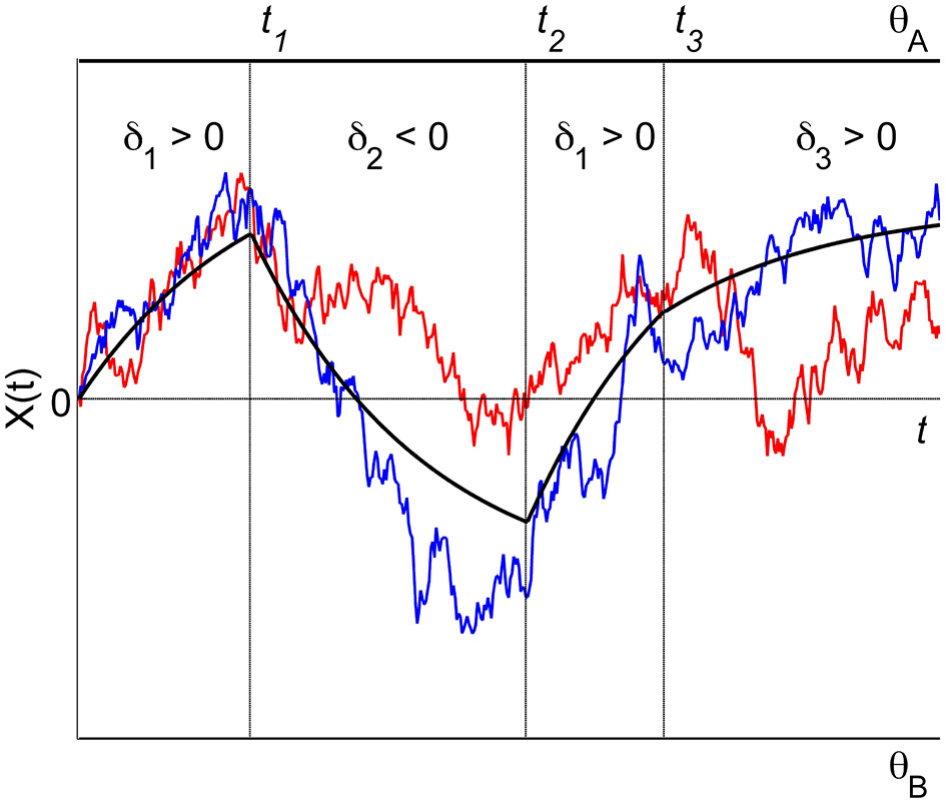
**A piecewise Ornstein-Uhlenbeck process with three different attributes.** The attribute order is (1, 2, 1, 3), attribute 1 is considered twice in the sequence of attribute consideration. Switching attention from one attribute to the next occurs at fixed times *t*_1_, *t*_2_, and *t*_3_. The trajectories reflect the accumulation process for two different trials. The black solid lines indicate the effective drift of the process.

For *K* attributes, each one to be considered for some specific time in some specific order it is convenient to introduce a formal schedule of both time and order. A finite time and order schedule consists of a set of *L* consecutive time intervals {[*t*_*l* − 1_, *t_l_*]}_*l*= 1, …,*L*_ and the attribute sequence {*k_l_* ∈ {1, …, *K*}}_*l*= 1, …,*L*_ which specifies that during the time interval [*t*_*l* − 1_, *t_l_*] the *k_l_*-th attribute is considered. At switching time *t_l_*, *l* = 1,…, *L* − 1, attention switches from attribute *k_l_* to attribute *k*_*l* + 1_. Depending on the situation, the final time *t_l_* may be set finite (then the decision process may also finish without deciding for one of the alternatives) or infinite. Consequently, the process *X*(*t*) determined by such a schedule is a piecewise Ornstein-Uhlenbeck process, defined over a finite partition *t*_0_ = 0 < *t*_1_ < … < *t*_*L* − 1_ < *t_L_* ≤ + ∞ of the time interval [0, *t_L_*], where for *t* ∈ [*t*_*l* − 1_, *t_l_*] the process is determined by (1) with *k* = *k_l_*. Figure 2 shows an example with three different attributes (*K* = 3) and a deterministic time and order schedule of length *L* = 4 with switching times *t_l_* independent of the trajectories, and attribute order (1, 2, 1, 3), i.e., *k*_1_ = 1, *k*_2_ = 2, *k*_3_ = 1, *k*_4_ = 3 (note that the first attribute is reconsidered once).

For fixed Δ resp. τ, the *m* × *m* transition probability matrix $\tilde{P}$*_n_* containing the transition probabilities $\tilde{P}$_*ii*′_: = *P*($\tilde{X}$_*n* + 1_ = *i*′|$\tilde{X}$_*n*_ = *i*) for the *n*-th step of the discrete-time random walk depends on the currently considered attribute defined by the time and order schedule, i.e., we set $\tilde{P}$*_n_* = *P_k_l__* if *n* = *n*_*l* − 1_, …, *n_l_* − 1, where *n*_0_ = 0, τ *n_l_* ≈ *t_l_* for *l* = 1, …, *L* (if *t_L_* = ∞, we formally set *n_L_* = ∞).

## 3. Choice probabilities and mean choice response times

In this section we derive the choice probabilities and mean choice response times for various time and order schedules. For simplicity we assume an unbiased process, i.e., with *X*(0) = 0 and symmetric decision thresholds, i.e., θ*_A_* = −θ*_B_*. Since the diffusion coefficient is a scaling parameter it will be set to σ = 1 for all attributes throughout. We start with the deterministic time and order schedule.

### 3.1. Deterministic time and order schedule

The evidence accumulation process for attribute *k*_1_, which is considered first, evolves until time *t*_1_ when the second attribute *k*_2_ comes into consideration, triggering a change in the accumulation process. This attribute in turn is considered until time *t*_2_ when a third attribute *k*_3_ is considered and so forth until a decision is initiated (or *t_l_* is reached). Let the random variables *T_A_* and *T_B_* denote the finite time when the process reaches a decision threshold θ*_A_* or −θ*_B_*, stops, and a decision response for *A* or *B* is initiated. With the switching times *t_l_* replaced by integers *n_l_* ≈ *t_l_*/τ, the choice probability *Pr*[choose *A*] = *Pr*(*T_A_* < ∞) is then approximated by the value *p_A_* obtained from the discrete random walk model as

(3) $$\begin{array}{l} Pr(T_{A}<\infty )\;\approx p_{A}:=Z^{\prime }\sum_{i=1}^{n_{1}}Q_{k_{1}}^{i-1}R_{A,k_{1}}\\ \;+\;Z^{\prime }Q_{k_{1}}^{n_{1}}\sum_{i=n_{1}+1}^{n_{2}}Q_{k_{2}}^{i-(n_{1}+1)}R_{A,k_{2}}+\ldots \ldots \\ \;+\;Z^{\prime }Q_{k_{1}}^{n_{1}}\ldots Q_{k_{L\;-\;1}}^{n_{L-1}-n_{L-2}}\sum_{i=n_{L-1}+1}^{n_{L}}Q_{n_{L}}^{i-(n_{L\;-\;1}+1)}R_{A,k_{L}}, \end{array}$$

where *Z* is the probability distribution for the initial state *X*(0). For instance, for an unbiased process, *Z* would be a coordinate vector with probability 1 at state 0 halfway between the decision thresholds. The remaining vectors and matrices are those defined in (2). The evidence accumulation process for a successive attribute starts with the final evidence state of the previous attribute. Note that *Z*′*Q*^*n*_1_^_*k*_1__ to *Z*′*Q*^*n*_1_^_*k*_1__…*Q*^*n*_*L* − 1_−*n*_*L* − 2_^_*k*_*L* − 1__ are defective distributions, i.e., the entries of these vectors do not sum up to 1, for the states of the random walk at discrete times *n*_1_,…,*n*_*L* − 1_. Further note that the stochastic process is time homogeneous within each time interval [0, *t*_1_) to [*t*_*L* − 1_, *t_l_*] but non-homogeneous across [0, *t_L_*] (see Diederich, 1992, 1995).

Similarly, the mean response time for choosing alternative *A* is approximated as

(4) $$\begin{array}{l} E[T_{A}|\text{choose}A]\approx ET_{A}:=\frac{\tau }{p_{A}}[Z^{\prime }\sum_{i=1}^{n_{1}}iQ_{k_{1}}^{i-1}R_{A,k_{1}}\\ \;+\;Z^{\prime }Q_{k_{1}}^{n_{1}}\sum_{i=n_{1}+1}^{n_{2}}iQ_{k_{2}}^{i-(n_{1}+1)}R_{A,k_{2}}+\ldots \ldots .\\ \;+Z^{\prime }Q_{k_{1}}^{n_{1}}\ldots Q_{k_{L-1}}^{n_{L-1}-n_{L-2}}\sum_{i=n_{L-1}+1}^{n_{L}}iQ_{n_{L}}^{i-(n_{L-1}+1)}R_{A,k_{L}}]. \end{array}$$

The probability and the mean response time for choosing alternative *B* can be determined similarly. Note that *p*_0_: = 1 − (*p_A_* + *p_B_*), the probability of not making a decision until the final time *t_L_*, is strictly positive if *t_L_* < ∞. As shown in Diederich (1997), these formulas can be further compactified. We will do this below for the general case of deterministic and random schedules by deriving an efficient recursion for their evaluation.

### 3.2. Random time and order schedule

The above derivation of formulas for choice probabilities and mean response times for a deterministic time and order schedule have counterparts for random schedules which we describe next in three steps.

#### 3.2.1. Random order schedule

For generating the attribute order {*k_l_*}_*l* = 1,…,*L*_, we consider stochastic *K* × *K* matrices *D*^(*l*)^ such that *d*^(*l*)^_*k*′*k*_ ≥ 0 describes the probability with which attention switches from the *k*′-th attribute to the *k*-th attribute at switching time *t_l_* ≈ τ *n_l_*, *l* = 1,…,*L* − 1. Normally, *d*^(*l*)^_*kk*_ = 0 would be assumed, to avoid a no switching situation. For two attributes *K* = 2, we must then have *d*^(*l*)^_11_ = *d*^(*l*)^_22_ = 0, *d*^(*l*)^_12_ = *d*^(*l*)^_21_ = 1, and the attribute sequence is either (1, 2, 1, 2, …) or (2, 1, 2, 1, …), depending on whether *k*_1_ = 1 or *k*_1_ = 2. For three attributes and *L* = 3, choosing

$$D^{\left(1\right)}=\left[\begin{array}{ccc} 0 & 1/2 & 1/2\\ 1/2 & 0 & 1/2\\ 1/2 & 1/2 & 0 \end{array}\right],\;D^{\left(2\right)}=\left[\begin{array}{ccc} 0 & 1 & 0\\ 1 & 0 & 0\\ 3/4 & 1/4 & 0 \end{array}\right],$$

would for *k*_1_ = 1 result in order sequences (1, 2, 1), (1, 3, 1), (1, 3, 2) with probability 1/2, 3/8, 1/8, respectively. The above matrix *D*^(1)^ models the situation when no preference or bias for considering attributes can be asserted.

#### 3.2.2. Random time schedule

We assume that the number of discrete time steps during which attention is paid to the *k*-th attribute is a discrete random variable denoted by *T_at_* with given distribution. In principle, this distribution may change its type and may have different parameters, such as expected value, depending on the attribute and the attribute order {*k_l_*}_*l* = 1, …, *L*_. This can be used to model time pressure and other temporal effects. However, often we assume one and the same distribution type for attention times across all attributes, and allow for different parameters only.

**Figure 3:**
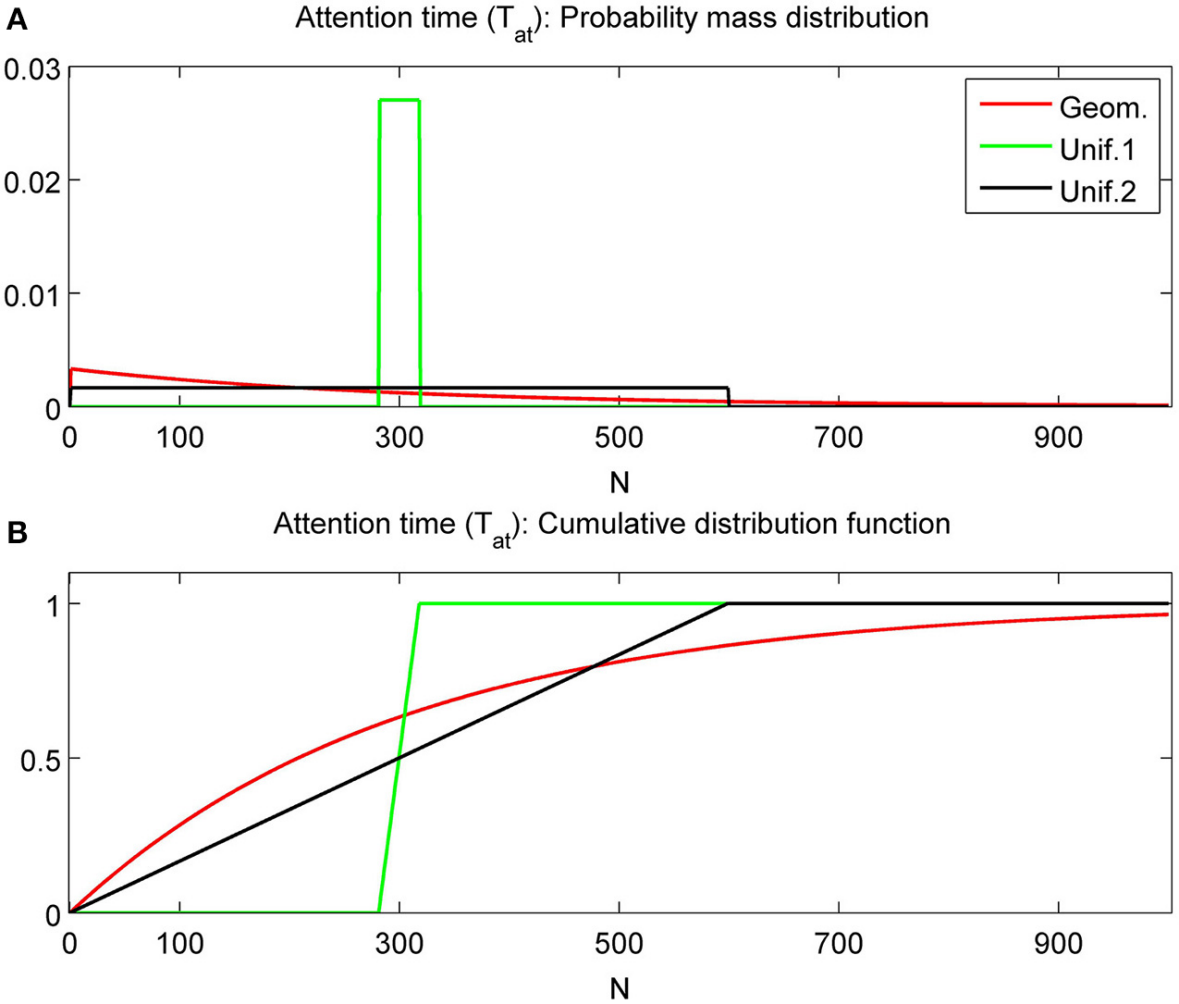
**Probability mass distributions (A) and cumulative distribution functions (B) for commonly used attention time distributions.** All distributions have expected value 300. The uniform distributions with *N* = 300 and *M* = *N*/2 = 150 are labeled as Unif.1 and with *N* = 300 and *M* = *N* − 1 = 299 as Unif.2. Geom. represents the geometric distribution.

For instance, the *geometric distribution* (as implicitly considered in Diederich, 1997) is given by

$$Pr(T_{at}=n)={\left(1-r\right)}^{n-1}r,\;n=1,2,\ldots ,$$

and characterized by a single parameter *r* > 0, with expectation 1/*r* and variance (1 − *r*)/*r*^2^, and the uniform distribution is defined as

$$Pr(T_{at}=n)=\frac{1}{2M+1},\;n=N-M,\ldots ,N+M,$$

with parameters *N* and *M* = 0, 1, …, *N* − 1 and expectation *N* and variance *M*(*M* + 1)/3. Details for other tested distributions (Poisson with parameter λ > 0, and binomial distributions with parameters *n* and *p*) are omitted. For comparable expectation values *E*(*T_at_*) (i.e., for parameter choices 1/*r* ≈ *N* ≈ λ ≈ *np*), the geometric distribution has much larger variance than the Poisson, binomial and uniform distribution with *M* ≈ $\sqrt{N}$ (the latter are very close to each other). Figure 3 shows the pdf and cdf for different *T_at_* distributions with fixed mean value *E*(*T_at_*) = 300. The two uniform distributions are with *M* = 150 = *N*/2 and *M* = 299 = *N* − 1. Varying the parameter *M* of the uniform distribution allows us to produce intermediate results between the deterministic and geometric distribution cases as shown in the following.

#### 3.2.3. Constructing random time and order schedules

We create a *random time and order schedule* of length *L* in two steps: First, given an initial distribution of *k*_1_ ∈ {1, …, *K*}, we create the attribute sequence {*k_l_*}_*l* = 2, …,*L*_ using a non-stationary Markov chain model with transition probability matrices *D*^(*l*)^, *l* = 1,…, *L* − 1. In a second step, for each *l* = 1,…,*L*, the attention time *T*^(*l*)^_*at*_ = *n_l_* − *n*_*l* − 1_ is created by the discrete random variable responsible for the attention time paid to the *k_l_*-th attribute, choices are independent for the different *l*. Consequently, *t_l_* − *t*_*l* − 1_ ≈ τ T^(*l*)^_*at*_ is the real attention time paid to the *k_l_*-th attribute. We note that *semi-random schedules*, where the sequence {*k_l_*} is given deterministically, and only the *T*^(*l*)^_*at*_ are determined as in the second step outlined above, are covered if we choose the *D*^(*l*)^ such that *d*^(*l*)^_*k_l_*,*k*_*l* + 1__ = 1.

To understand the recursive computation of choice probabilities and mean response times in this more general case, we first consider the special cases *L* = 1, 2, and illustrate the derivation on some distribution types of the random variable *T_at_* generating attention times by providing concrete formulas. In general, the distribution for *T_at_* is given by its probability mass distribution (pdf) and cumulative distribution function (cdf)

(5) $$\begin{array}{l} Pr(T_{at}=n)=p_{n,k},\\ Pr(T_{at}\leq n)=f_{n,k}:=\sum_{i=0}^{n}p_{i,k},\;n=0,1,\ldots . \end{array}$$

We start with *L* = 1, and will drop the index *l* from the notation introduced in the previous subsection. Since the probability of choosing alternative *A* at the *i*-th step is given by *Z*′*Q_k_*^*i*−1^*R_*A*,*k*_*, *i* = 1, …, *T_at_*, and *T_at_* is a random variable distributed according to (5) we get

$$\begin{array}{l} p_{A,k}=\sum_{n=1}^{\infty }p_{n,k}Z^{\prime }\left(\sum_{i=1}^{n}Q_{k}^{i-1}\right)R_{A,k}\\ \;=Z^{\prime }\left[\sum_{i=1}^{\infty }\left(\sum_{n=i}^{\infty }p_{n,k}\right)Q_{k}^{i-1}\right]R_{A,k}\\ \;=Z^{\prime }\left[\sum_{i=0}^{\infty }\left(1-f_{i,k}\right)Q_{k}^{i}\right]R_{A,k}. \end{array}$$

A similar formula holds for *p*_*B*,*k*_. To avoid repetition, introduce the row vector *p*_*AB*,*k*_: = [*p*_*B*,*k*_, *p*_*A*,*k*_], then

(6) $$p_{AB,k}=Z^{\prime }V_{k},\;V_{k}:=\left[\sum_{i=0}^{\infty }\left(1-f_{i,k}\right)Q_{k}^{i}\right]R_{k}.$$

The 2 × (*m* − 2) matrix *V_k_* depends on the attribute and its parameters via *Q_k_*, *R_k_*, and on the chosen attention time distribution and the cdf (*f*_*n*,*k*_). For the discussed concrete attention time distributions these matrices may be precomputed, in some cases closed-form expressions can be found, e.g., for the geometric distribution with parameter *r* = *r_k_* we have

$$\begin{array}{l} V_{k}=\sum_{i=0}^{\infty }\left(\sum_{j=i+1}^{\infty }r_{k}{\left(1-r_{k}\right)}^{j-1}\right)Q_{k}^{i}R_{k}\\ \;=\sum_{i=0}^{\infty }{\left(1-r_{k}\right)}^{i}Q_{k}^{i}R_{k}={\left(I-\left(1-r_{k}\right)Q_{k}\right)}^{-1}R_{k}. \end{array}$$

Next we discuss choice probabilities for the case *L* = 2, assuming for simplicity that the attention time distribution is the same for all attributes. To save on indices, denote *k*_1_ ≡ *k*′, *k*_2_ ≡ *k*, and *D*^(1)^ ≡ *D* (this matrix is responsible for the random choice of *k* given any *k*′). Then the decision probability vector *p*_*AB*,*k*′, *k*_ for reaching alternatives *B* or *A* in with attribute order (*k*′,*k*) has two parts: the probabilities of having decided on while still considering the *k*′-th attribute (i.e., *T_A_*/τ ≤ *T*′_*at*_, where *T*′_*at*_ is the randomly generated attention time for the first attribute *k*′) plus the probabilities that τ *T*′_*at*_ < *T_A_*/τ ≤ *T*′_*at*_ + *T_at_*, where *T_at_* is the randomly (and independently) generated attention time for the second attribute *k*. On top of this, *k* itself is randomly chosen according to the entries in the *k*′-th row of *D*. Thus, for each fixed *k*_1_ = *k*′ and *n*_1_ = *T*′_*at*_ according to (6) probabilities for reaching a decision after *n*_1_ are given by

$$\begin{array}{l} {\left[Pr(T_{at^{\prime }}<\frac{T_{B}}{\tau }<\infty ),Pr(T_{at^{\prime }}<\frac{T_{A}}{\tau }<\infty )\right]}_{n_{1}=T_{at^{\prime }},k_{1}=k^{\prime }}\\ \;\approx \sum_{k=1}^{K}d_{k^{\prime }k}Z^{\prime }Q_{k^{\prime }}^{n_{1}}V_{k}=Z^{\prime }Q_{k^{\prime }}^{T_{at^{\prime }}}(\sum_{k=1}^{K}d_{k^{\prime }k}V_{k}). \end{array}$$

Thus, for *L* = 2, the choice probabilities (under the assumption that *k*_1_ = *k*′ is fixed) can be obtained as

$$\begin{array}{l} {\left[p_{B},p_{A}\right]}_{k_{1}=k^{\prime }}=Z^{\prime }V_{k^{\prime }}+\sum_{n\geq 0}p_{n,k^{\prime }}Z^{\prime }Q_{k^{\prime }}^{n}\left(\sum_{k=1}^{K}d_{k^{\prime }k}V_{k}\right)\\ \;=Z^{\prime }[V_{k^{\prime }}+(\sum_{n\geq 0}p_{n,k^{\prime }}Q_{k^{\prime }}^{n})(\sum_{m=1}^{M}d_{k^{\prime }k}V_{k})]\\ \;=Z^{\prime }[V_{k^{\prime }}+B_{k^{\prime }}(\sum_{k=1}^{K}d_{k^{\prime }k}V_{k})],\;k^{\prime }=1,\ldots ,K, \end{array}$$

where

(7) $$B_{k}=\sum_{n\geq 0}p_{n,k}Q_{k}^{n},\;k=1,\ldots ,K,$$

are (*m* − 2)× (*m* − 2) matrices depending on the attribute and attention time distribution type. For example, for the geometric distribution this simplifies to *B_k_* = *r_k_Q_k_*(*I* − (1 − *r_k_*)*Q_k_*)^−1^, closed form expressions are available for Poisson, binomial, and uniform distributions as well.

For arbitrary *L*, it is more convenient to write the resulting recursion in terms of block-matrix-vector operations. Denote by

**Table:** 

**Z**	the *K* × 1 array with each entry equal to the initial distribution *Z* (and think of **Z**′ as its transpose, a 1× *K* array with entries *Z*′),
**B**	the *K* × *K* diagonal array with the *B_k_* on the diagonal (similarly for **C** defined later),
**I**	the *K* × *K* diagonal array, with identity matrices *I* of the appropriate size on the diagonal,
**V**	the *K* × 1 array with the *V_k_* as entries (similarly for **W** defined later), and
**p**_*AB*_	the *K* × 2 matrix, whose rows are the choice probabilities [*p_A_*, *p_B_*]|_*k*_1_ = *k*_ defined before in the case *L* = 2.

Then the above result for *L* = 2 can be compactly written as

(8) $$p_{AB}=Z^{\prime }(I+BD)V.$$

Note that the product **B***D* of the array **B** with the matrix *D* is interpreted as the *K* × *K* array with *d*_*k*′*k*_*B*_*k*′_ as entry in row *k*′ and column *k*. Moreover, by iterating (8), one arrives at the formula for arbitrary *L*:

(9) $$p_{AB}=Z^{\prime }(I+BD^{\left(1\right)})\ldots (I+BD^{\left(l-1\right)})V.$$

Formulas for mean response times can be derived similarly. Indeed, for *L* = 1, denote by *ET*_*A*,*k*_ the mean response time for reaching alternative *A* when considering the *k*-th attribute for a random time *T_at_* distributed according to (5). Then *ET*_*A*,*k*_ ≈ τ *et*_*A*,*k*_/*P*_*A*,*k*_, where

(10) $$\begin{array}{l} et_{A,k}=\sum_{n=1}^{\infty }p_{n,k}(\sum_{i=0}^{n-1}\left(i+1\right)Z^{\prime }Q_{k}^{i})R_{A,k}\\ \;=Z^{\prime }\left[\sum_{i=0}^{\infty }\left(\sum_{n=i+1}^{\infty }p_{n,k})(i+1\right)Q_{k}^{i}\right]R_{A,k}\\ \;=Z^{\prime }\left[\sum_{i=0}^{\infty }\left(1-f_{i,k})(i+1\right)Q_{k}^{i}\right]R_{A,k}. \end{array}$$

Similarly for *ET*_*B*,*k*_ and *et*_*B*,*k*_. Thus, similar to (6), we can write

(11) $$\begin{array}{l} et_{AB,k}:=[et_{B,k},et_{A,k}]=Z^{\prime }W_{k},\\ \;W_{k}:=\left[\sum_{i=0}^{\infty }\left(1-f_{i,k})(i+1\right)Q_{k}^{i}\right]R_{k},\;k=1,\ldots ,K. \end{array}$$

The matrices *W_k_* can be precomputed to any accuracy at essentially the same cost as the *V_k_*. For particular distributions, the formulas can be turned into closed form expressions.

Next, let us look at *L* = 2. By using similar notation and arguments as for choice probabilities, the quantities *et*_*A*,*k*′,*k*_, *et*_*B*,*k*′,*k*_ have a part before and after *T*′_*at*_. This, together with (10), (11), gives

$$\begin{array}{l} et_{AB}{|}_{k_{1}=k^{\prime }}=Z^{\prime }W_{k^{\prime }}+\sum_{n=0}^{\infty }p_{n,k}Z^{\prime }Q_{k^{\prime }}^{n}\left(\sum_{k=1}^{K}d_{k^{\prime }k}(nV_{k}+W_{k})\right)\\ \;=Z^{\prime }[W_{k^{\prime }}+\left(\sum_{i=0}^{\infty }p_{i,k^{\prime }}iQ_{k^{\prime }}^{i}\right)\left(\sum_{k=1}^{K}d_{k^{\prime }k}V_{k}\right)\\ \;+\left(\sum_{i=0}^{\infty }p_{i,k^{\prime }}Q_{k^{\prime }}^{i}\right)\left(\sum_{k=1}^{K}d_{k^{\prime }k}W_{k}\right)]\\ \;=Z^{\prime }\left[W_{k^{\prime }}+C_{k^{\prime }}\left(\sum_{k=1}^{K}d_{k^{\prime }k}V_{k}\right)+B_{k^{\prime }}\left(\sum_{k=1}^{K}d_{k^{\prime }k}W_{k}\right)\right], \end{array}$$

where

(12) $$C_{k}=\sum_{n\geq 0}p_{n,k}nQ_{k}^{n},\;k=1,\ldots ,K.$$

Thus, the counterpart of (8) is

(13) $$et_{AB}=Z^{\prime }((CD)V+(I+BD)W),$$

From here, combining with (8), a joint recursion for computing **p**_*AB*_ and **et**_*AB*_ results:

(14) $$\begin{array}{l} {}[p_{AB},et_{AB}]=[Z^{\prime },Z^{\prime }]\left[\begin{array}{cc} I+BD^{\left(1\right)} & 0\\ CD^{\left(1\right)} & I+BD^{\left(1\right)} \end{array}\right]\ldots \\ \;\left[\begin{array}{cc} I+BD^{\left(L-1\right)} & 0\\ CD^{\left(L-1\right)} & I+BD^{\left(L-1\right)} \end{array}\right]\left[\begin{array}{c} V\\ W \end{array}\right]. \end{array}$$

We conclude this section with a few remarks. In Diederich (1997), under the name MADD/pp, a slightly different presentation of random schedules is given for the special case of geometrically distributed attention times. It is not hard to see, that (with the notation *r_ij_* used in the *K* = 3 example presented in Section 4.2 Diederich, 1997) our model is equivalent to MADD/pp as *L* → ∞, if we set *r_k_* = 1 − *r_kk_* for the parameters *r* of the geometrically distributed *T_at_*, *k* = 1, 2, 3, and *d_kk_* = 0, *d*_*kk*′_ = *r*_*kk*′_/(1 − *r_kk_*), *k*′≠ *k*, for the entries of the matrix *D* = *D*^(*l*)^, *l* ≥ 1. The advantage of the MADD/pp model is that it provides closed form formulas for the case *L* = ∞, a possibility that we did not pursue here for other types of attention time distributions.

In previous sequential decision models with finite *L* (Diederich, 1997), the last attribute was always considered infinitely long (infinite decision horizon) to avoid the situation of no decision, i. e., *p*_0_ > 0. This can be incorporated into the current model by modifying the definition of the matrices *V_k_*, *W_k_* corresponding to the last interval [*t*_*L* − 1_,∞) to

$$V_{k}={\left(I-Q_{k}\right)}^{-1}R_{k},\;W_{k}={\left(I-Q_{k}\right)}^{-2}R_{k},\;k=1,\ldots ,K,$$

and modifying the recursion (14) slightly. Alternatively, one can artificially change the parameters of the attention time distribution for *l* = *L* such that its expected value is sufficiently large, and make *p*_0_ practically negligible. Since infinite decision horizons do not seem to adequately reflect the situation of a real decision process or laboratory experiment, it might be interesting to work under scenarios where *t_l_* is fixed and finite that we described in this paper.

## 4. Simulations

We present some simulations that demonstrate the predictive power of the proposed model. We focus on features that have not been considered in Diederich (1997) for the deterministic case. Throughout this section we fix certain parameters, such as σ = 1, θ*_A_* = −θ*_B_* = 10, $\Delta =\frac{1}{4},\tau =\frac{1}{16}$ (this implies a state space size of *m* = 81), and always start at the neutral position *X*(0) = 0 between choice alternatives *A* and *B*.

### 4.1. Impact of attention time distributions

**Figure 4:**
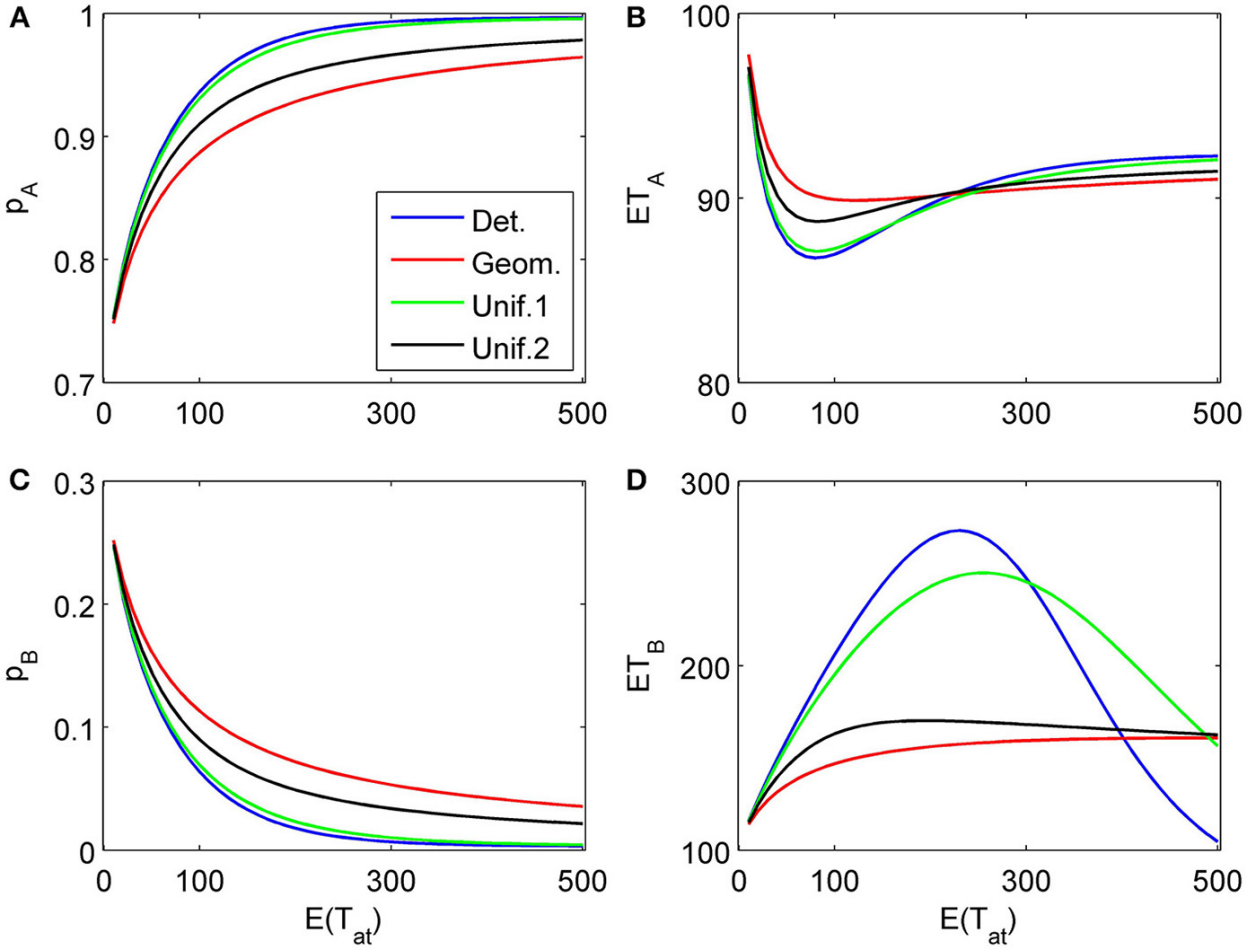
**Choice probabilities **(A,C)** and mean response times **(B,D)** as functions of the expected attention time *E*(*t*_1_) = 10… 500 paid to the first attribute for different distribution types.** The attribute considered first for a random time *t*_1_ strongly favors alternative *A*, followed by a second attribute which only weakly favors *A* but is considered indefinitely. Note that graphs for distribution types with small variance are almost indistinguishable from the graph corresponding to deterministically fixed *t*_1_ (variance 0) and therefore are omitted here.

**Figure 5:**
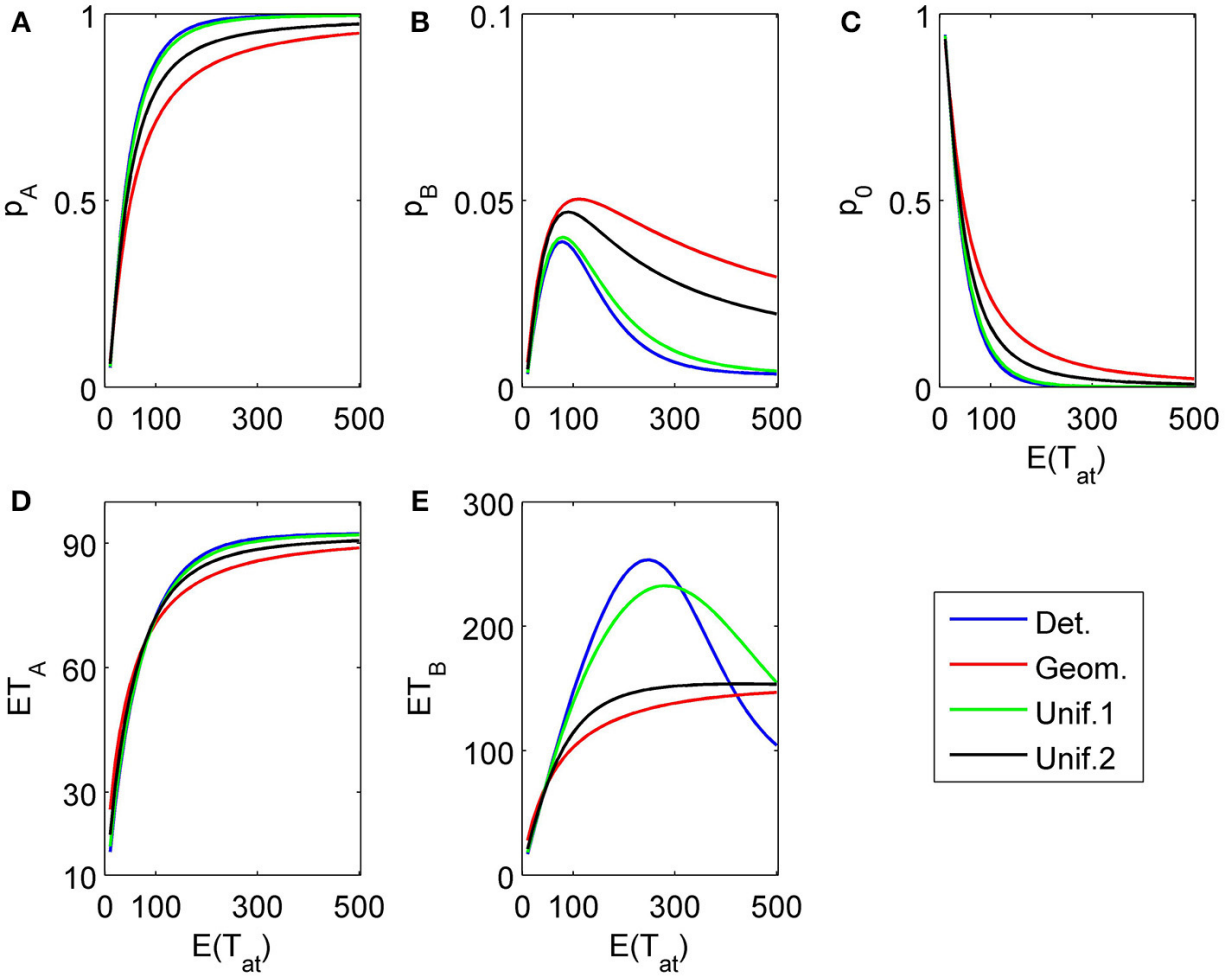
**Same as in Figure 4 but now the second attribute is also considered for a random finite time *t*_2_ − *t*_1_ whose distribution is the same as for *t*_1_ [in particular, *E*(*t*_2_ − *t*_1_) = *E*(*t*_1_)]. (A)** and **(B)** show the choice probabilities for choosing alternative A and B, respectively. **(C)** shows the probability *p*_0_ of not reaching a decision which naturally decays if the expected attribute attention time grows. **(D)** and **(E)** show the expected mean response times for choosing alternative A and B, respectively, as functions of the expected attention time *E*(*t*_1_) = 10… 500 paid to the first attribute for different distribution types.

First, we show how different assumptions on the randomness of the attention time *T_at_* (i.e., the time spent on considering a certain attribute) influences choice probabilities and mean response times. In the first example, we assume just two attributes with parameters δ_1_ = 0.2, γ_1_ = 0.03, δ_2_ = 0.04, γ_2_ = 0.003, both attributes favor alternative *A*, the first one more strongly than the second one^2^. The attributes are considered only once (*L* = 2), with order *k*_1_ = 1, *k*_2_ = 2. The first attribute is considered for time *t*_1_ = τ *n*_1_, where *n*_1_ is a random variable *T_at_* described above with given expectation *N*. For the second attribute we compare two situations: (1) We assume an infinitely long decision horizon *t*_2_ = ∞, and (2) we determine a finite time horizon *t*_2_ = τ *n*_2_ by choosing *n*_2_ = *n*_1_ + *T_at_* which is also *T_at_* distributed with the same expected value *N*. These two situations are depicted in Figures 4, 5. The graphs show choice probabilities and mean response times as functions of the expectation τ *E*(*T_at_*) of the real attention times. Lines of different color represent different distributions. Distributions with a small variance, such as the Poisson distribution, the binomial distribution, and the uniform distribution with *M* ≈ $\sqrt{N}$ produce results indistinguishable from the deterministic case. This holds for all tested situations shown below. This means, small uncertainties in attention time spans do not influence the observable choice frequencies and mean response times. However, as the variance of the attention times grows, we see quantitative and qualitative changes. Compared to the deterministic attention time situation, the geometric distribution differs most, and the uniform distributions with *M* = *N*/2 = 150 (Unif.1) and *M* = *N* − 1 = 299 (Unif.2) are intermediate. Moreover, there is expectedly a big difference for small mean attention times between finite and infinite decision horizons. Most importantly, for the former case it predicts a probability *p*_0_ > 0 of not deciding within the available time *t*_2_. We claim that for many situations, where an infinite time horizon does not represent reality well enough, our finite schedule model might be more appealing. This aspect will be pursued in further research.

**Figure 6:**
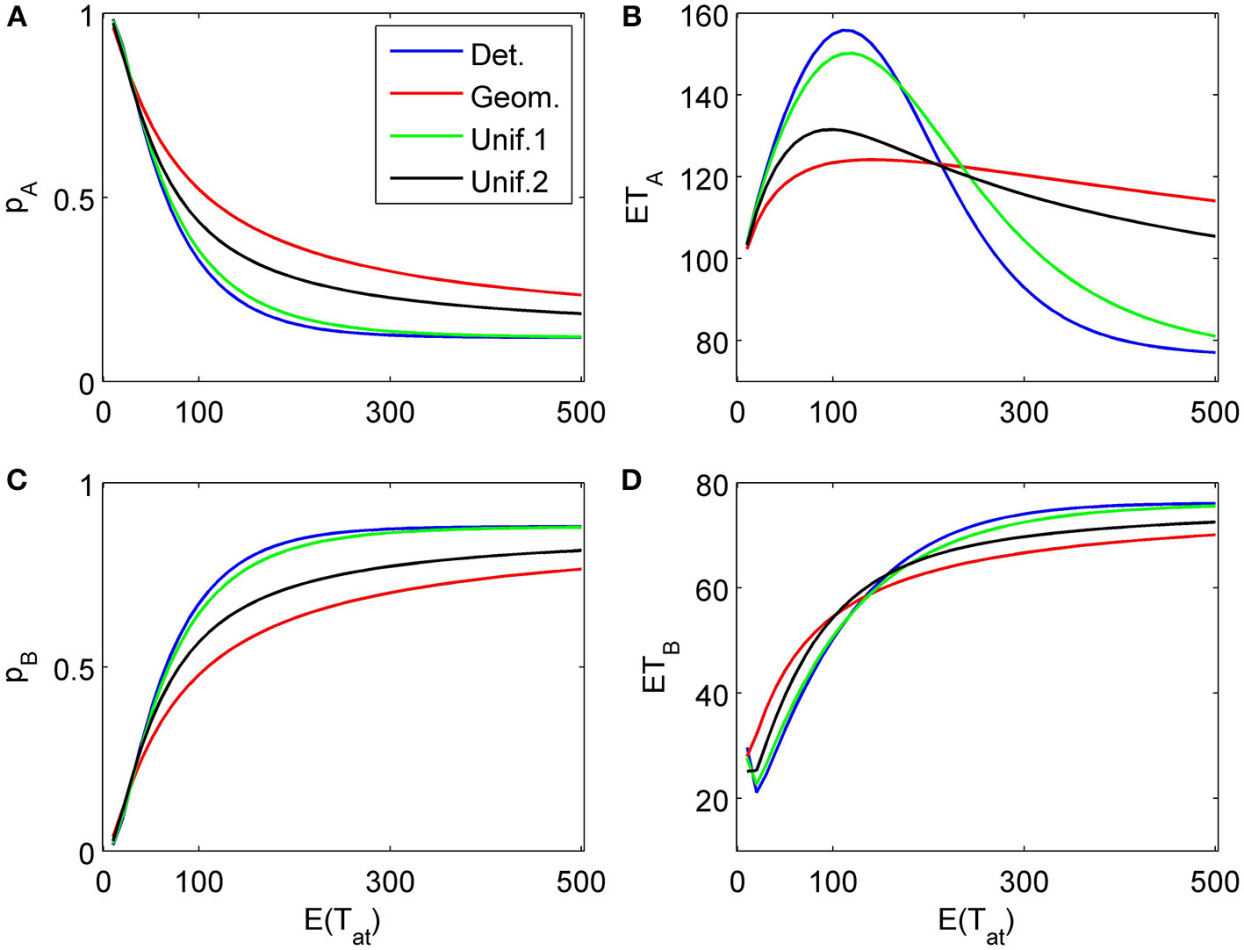
**Choice probabilities (A,C) and mean response times (B,D) for a decision situation where an attribute favoring alternative *B* is considered first for a random time *t*_1_, followed by a second attribute strongly favoring *A* but considered indefinitely.** We show graphs of choice probabilities and mean response times as functions of the expected attention time *E*(*t*_1_) = 10… 500 paid to the first attribute for different distribution types. Again, graphs for distribution types with small variance are indistinguishable from each other.

**Figure 7:**
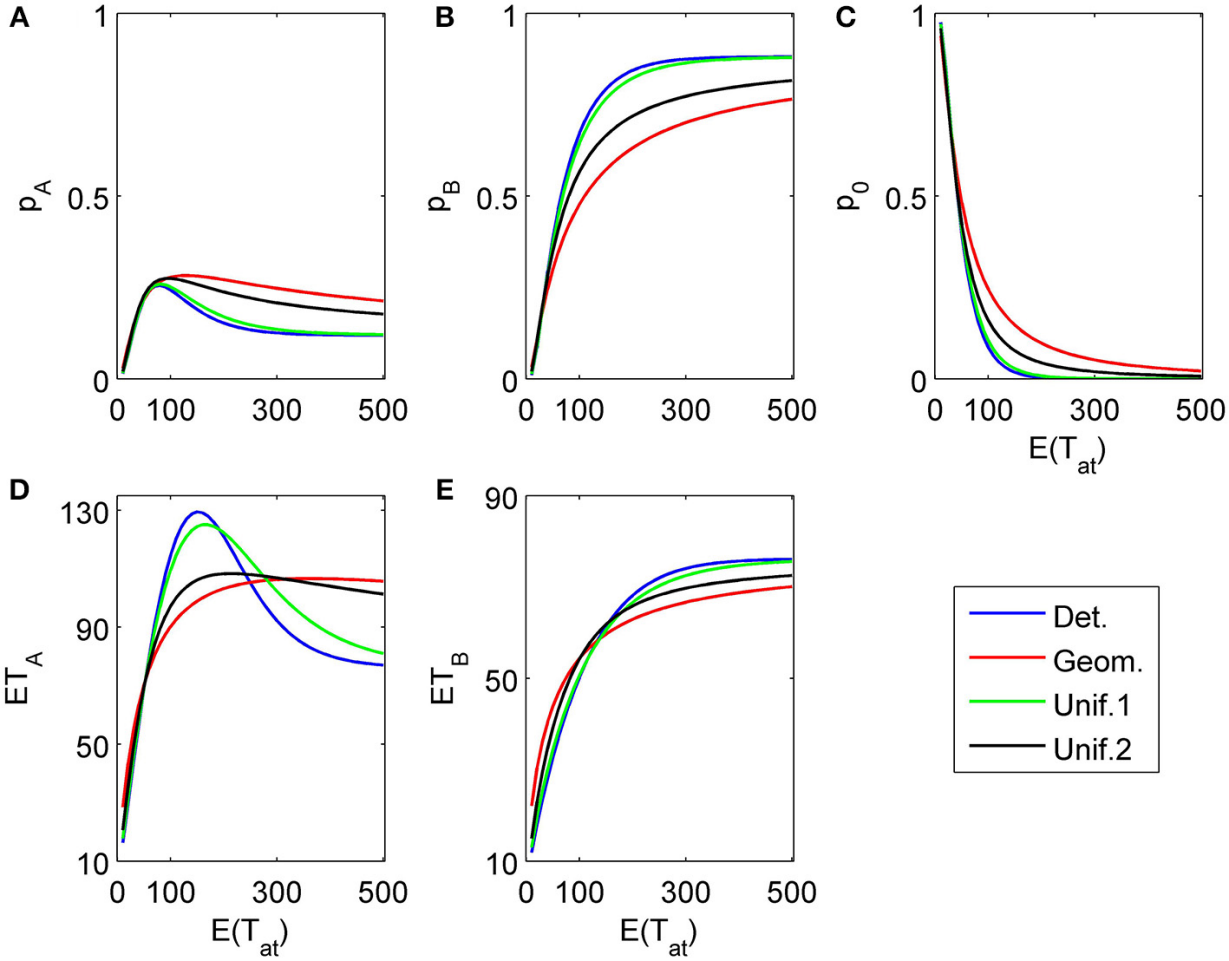
**Same as in Figure 6 but now the second attribute is also considered for a random finite time *t*_2_ − *t*_1_ whose distribution is the same as for *t*_1_. (A)**, **(B)**, and **(C)** show the choice probabilities for choosing alternatives A, B and none, respectively. **(D)** and **(E)** show the mean response times for choosing alternatives A and B, respectively.

Figures 6, 7 show similar simulation results for the situation of considering first an attribute favoring *B* (δ_1_ = −0.1, γ_1_ = 0) followed by an attribute more strongly favoring *A* (δ_2_ = 0.2, γ_2_ = 0.03). As expected, the results look now different, however, the main conclusions from the previous example concerning the influence of the randomness type for attention times and the differences for finite vs. infinite time horizons remain the same. Most importantly here, the model predicts a preference reversal (i.e., choice probabilities from below 0.5 to above 0.5) as a function of attention time when one attribute is in favor of choosing alternative A and the other in favor of choosing alternative B. Parameter studies, as in Diederich (1997), will be pursued further elsewhere.

**Figure 8:**
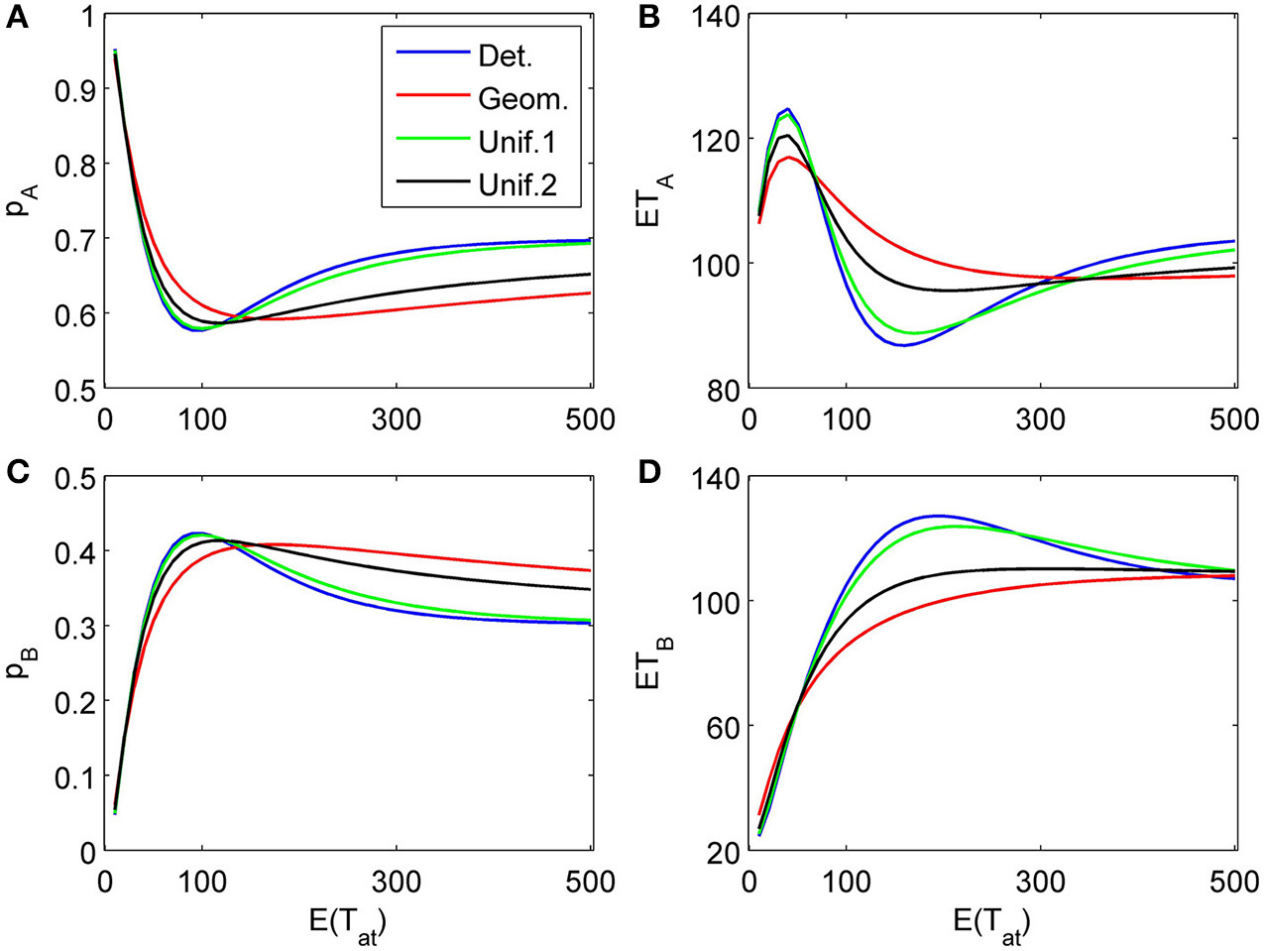
**Choice probabilities (A,C) and mean response times (C, D) for a decision model with three attributes.** An attribute weakly favoring alternative *A* is considered first for a random time *t*_1_, followed by a second attribute favoring *B* considered for a random time *t*_2_ − *t*_1_, while the last attribute (strongly favoring *A*) is considered indefinitely. The random attention times *t*_1_ and *t*_2_ − *t*_1_ for the first two attributes are independently chosen from the same distribution. We show graphs of choice probabilities and mean response times as functions of the expected attention time *E*(*t*_1_) = *E*(*t*_2_ − *t*_1_) = 10… 500 for different distribution types. Again, small variance distributions yield almost identical results.

To complete the picture, we show a three-attribute example (*K* = 3) in Figure 8. The chosen attribute parameters are now δ_1_ = 0.04, γ_1_ = 0.003, δ_2_ = −0.1, γ_2_ = 0, δ_3_ = 0.2, γ_3_ = 0.03, i.e., a weakly in favor of *A*, in favor of *B*, and strongly in favor of *A* sequence of attributes. Attention times for the first two attributes are chosen independently from each other but with the same distribution with fixed mean value; the last attribute is considered indefinitely.

### 4.2. Dependence on attribute order

**Figure 9:**
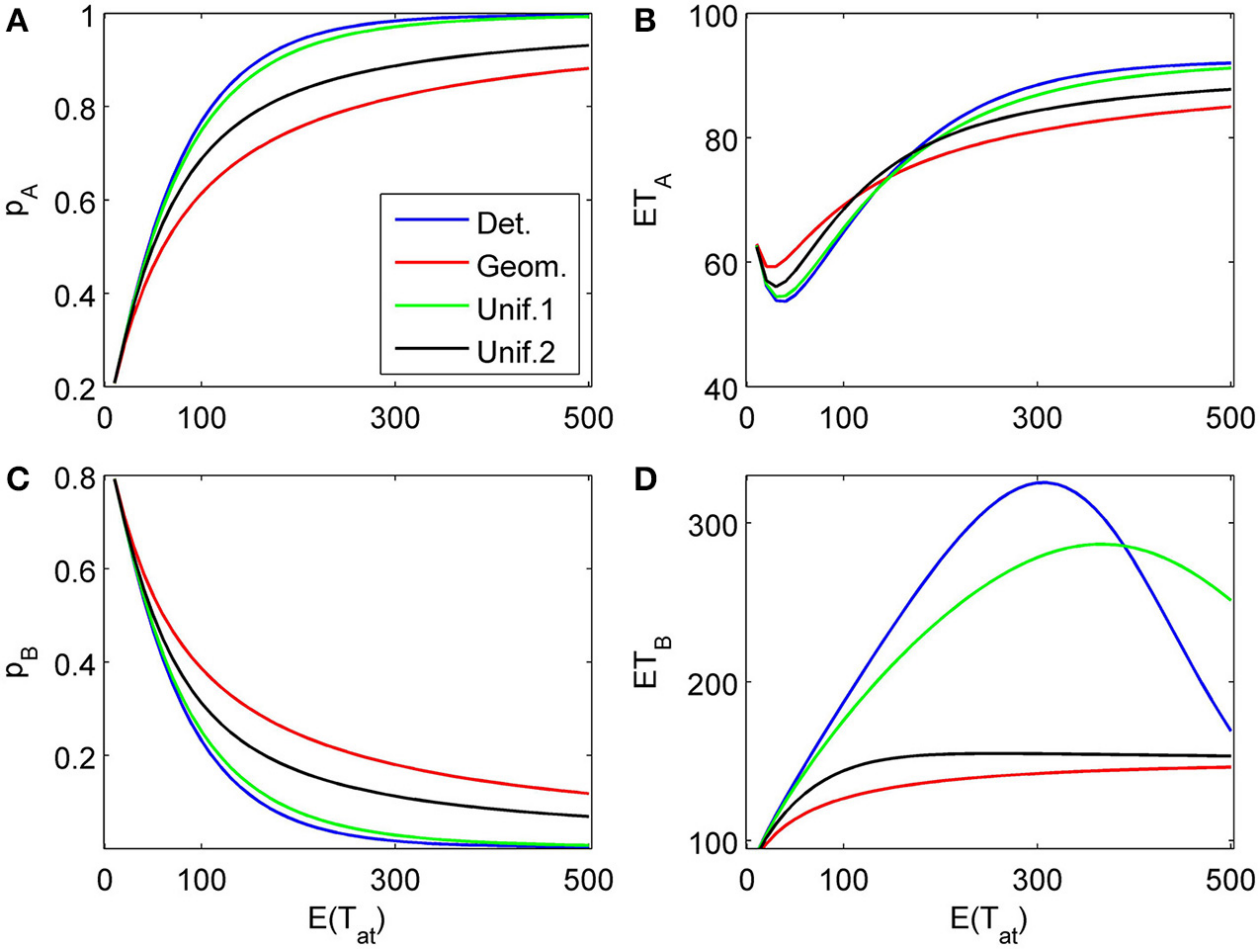
**Same as in Figure 6 but with a different attribute order: First the attribute strongly in favor of *A* is considered for a finite random time *t*_1_, then the attribute favoring *B* is considered indefinitely long. (A)** and **(C)** show the choice probabilities for choosing alternatives A and B respectively. **(B)** and **(D)** show the mean response times for choosing alternatives A and B, respectively.

**Figure 10:**
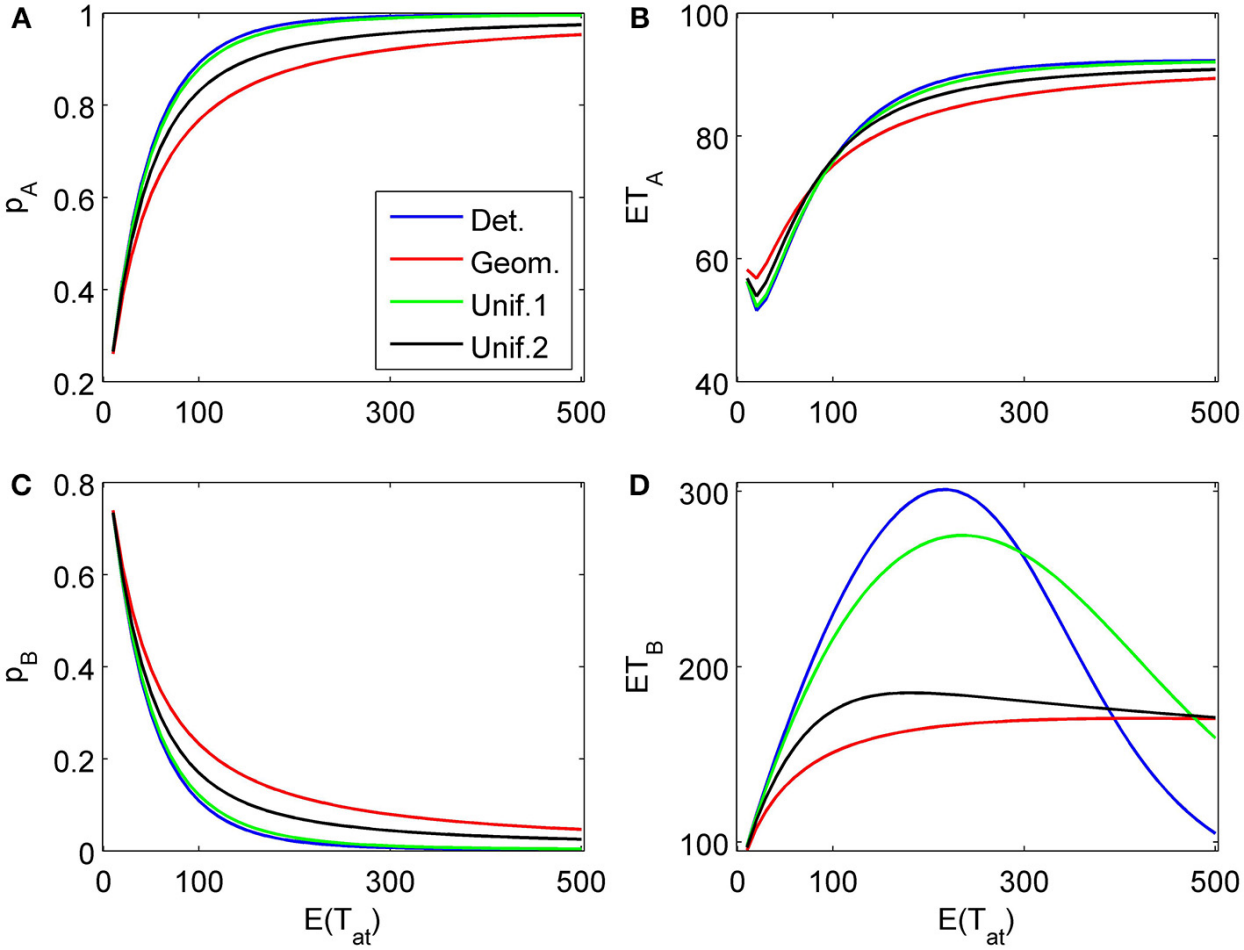
**Same as in Figure 8 but with a different attribute order: First the two attributes in favor of *A* (strong followed by weak) are considered for finite random periods of time, then the attribute favoring *B* is considered indefinitely long. (A)** and **(C)** show the choice probabilities for choosing alternatives A and B, respectively. **(B)** and **(D)** show the mean response times for choosing alternatives A and B, respectively.

**Figure 11:**
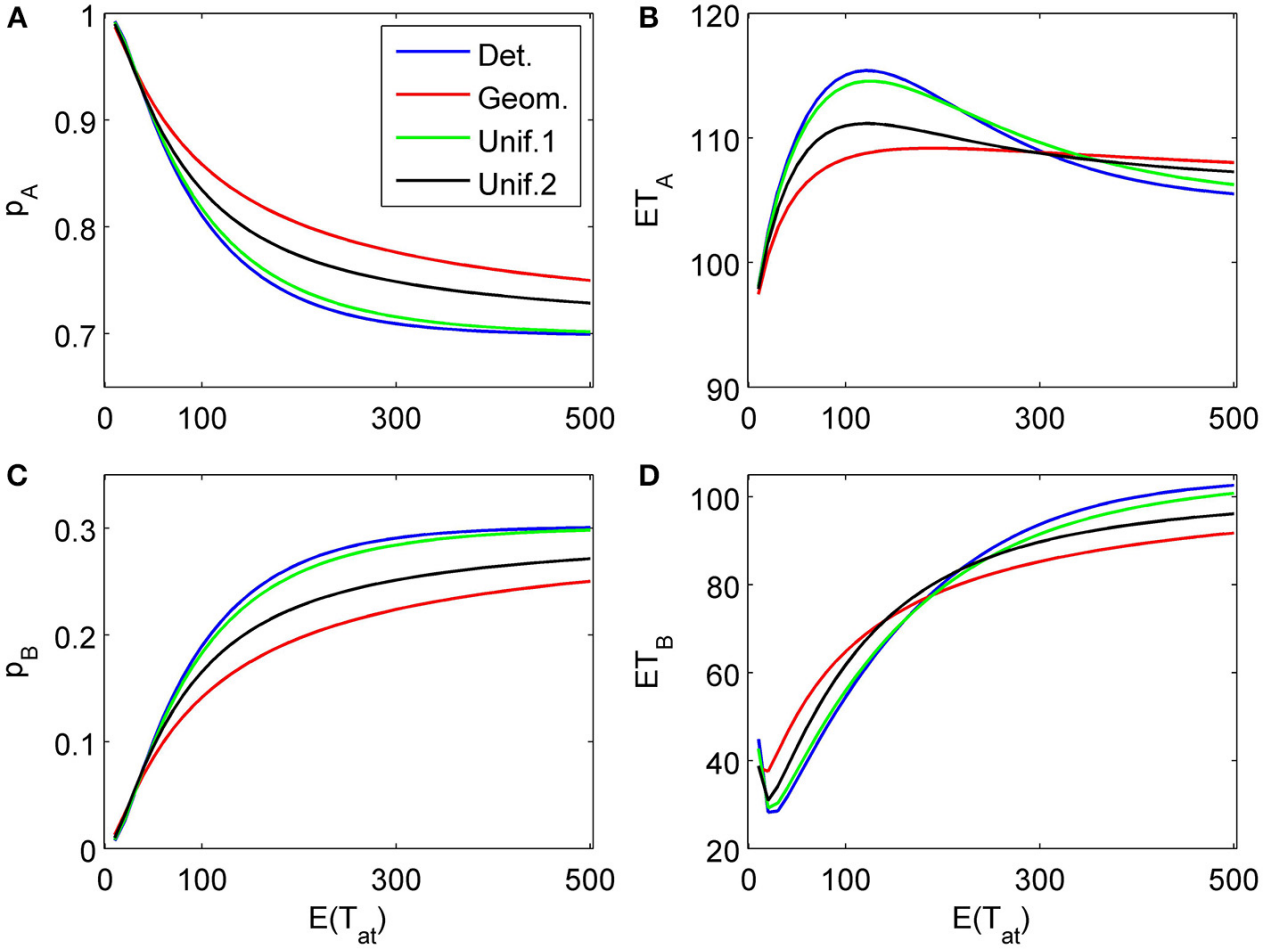
**Same as in Figure 4 but with a different attribute order: The attribute considered first for a random time *t*_1_ weakly favors alternative *A*, followed by a second attribute which strongly favors *A* but is considered indefinitely. (A)** and **(C)** show the choice probabilities for choosing alternatives A and B respectively. **(B)** and **(D)** show the mean response times for choosing alternatives A and B, respectively.

The proposed sequential decision model is sensitive to the order in which the attributes are consider. If we consider in the aforementioned second two-attribute example the attribute in favor of *A* first, and then the attribute in favor of *B* we get very different patterns as shown in Figure 9 compared to Figure 6. A similar effect is true for the above *K* = 3 example. In Figure 10, the attribute in favor of *B* is now the last one; the graphs need to be compared with Figure 8. One interesting pattern can be observed. If the evidence for choosing one alternative decreases in the sequence of attribute consideration then the model predicts faster choice response times for the more frequently chosen alternative—a typical pattern observed in response time analysis. However, if the evidence increases in the sequence of attribute consideration then the model predicts faster choice response times for the less frequently chosen alternative which has been called *fast error*, as shown in Figure 11 compared to Figure 4. Simply by changing the order of attribute processing the model predicts a complex pattern of choice response times and choice probabilities.

So far, all examples shown are with a fixed, deterministic attribute order with no repetitions (semi-random schedule, *L* = *K*). The evaluation of fully random time and order schedules requires larger *L*, and will be presented elsewhere.

## 5. Concluding remarks

The proposed *multiattribute attention switching* (MAAS) model can predict a very complex choice probability/(mean) choice response time pattern. It may appear too flexible to be testable. However, this is not the case. If two attributes both favor alternative, *A* say, and the first attribute that is considered provides more evidence for choosing *A* than the second (δ_1_ > δ_2_), then the model predicts always shorter response times for the more frequently chosen alternative, here *A*, regardless of the assumed underlying attention time distribution. If the order of processing these attributes is reversed, i.e., the attribute that favors alternative *A* less is considered first (δ_2_ > δ_1_), then the model always predicts faster responses for the less frequently chosen alternative, here *B*, again regardless of the assumed underlying attention time distribution. A single stage process can only account for this pattern by assuming variability in starting positions and variability in drift rates, i.e., a statistical means where the drift rate itself is a random variable. It is difficult experimentally to disentangle the variability stemming from the stochastic process itself and the variability from the distribution of different drift rates. As Jones and Dzhafarov (2013) pointed out, the predictions of various sequential sampling models rest upon the assumptions made about the assumed probability distributions. This is not the case here. The model is falsifiable without assuming specific distributions. Rather than relying on statistical mechanisms to ensure an observed response patterns we rely on assumptions about cognitive processes such as attention switching and salience. The specific attention time distribution used for an application may be related to the experimental paradigm. For instance, when tracking eye movements, the sequence of attribute consideration and the switching times are directly observable, and a deterministic or a uniform distribution with a small variance is advisable. When all attributes are shown simultaneously, like in complex objects, and attention may shift at any moment in time a geometric distribution or a uniform distribution with a large variance may describe the situation better. Testing the model rigorously will be pursued in the future.

### Conflict of interest statement

The authors declare that the research was conducted in the absence of any commercial or financial relationships that could be construed as a potential conflict of interest.

## Footnotes

^1^The notion of *attributes* is defined here in a broad sense. For example, it includes dimensions such as color and size of visual target; amplitude and frequency of a tone; different modalities in a crossmodal task; payoff information and perceptual information; attitudinal evidence and perceptual evidence; prize and quality of a consumer product and more.

^2^Note that when looking only at the numerical values of the drift parameter δ_1_ = 0.2 and the decision criterion θ*_A_* = 10 and assuming that the attention times *t*_1_ to the first attribute are large enough it would suggest mean response times in the range *T_A_* ≈ 50 (and very small *p_B_*). However, since γ_1_ = 0.03 it leads to a negative effective drift δ_1_ − γ_1_*X*(*t*) if *X*(*t*) comes close θ*_A_*, and the mean response times become much longer. This also demonstrates the effect of the parameter γ*_k_*, and a difference between Ornstein-Uhlenbeck process and Wiener process based models.
